# Fluid accumulation increases the risk of AKI progression and death in critically ill patients with early AKI

**DOI:** 10.1186/cc13561

**Published:** 2014-03-17

**Authors:** MR Raimundo, S Crichton, Y Syed, J Martin, M Ostermann

**Affiliations:** 1 Hospital Beatriz Ângelo, Loures, Portugal; 2 Guy's & St Thomas Hospital, London, UK

## Introduction

Fluid therapy is a cornerstone in the management of patients with evolving acute kidney injury (AKI). Despite the wide availability of advanced hemodynamic monitoring to guide therapy, there are few data on how to manage hemodynamics optimally once early AKI has occurred. Our aim was to investigate the association between cumulative fluid balance (FB)/fluid administration and outcome (progression to AKI III and hospital mortality) in critically ill patients with early AKI, and its interaction with other hemodynamic parameters.

## Methods

A retrospective analysis (2-year period) of all patients admitted to an adult ICU with AKI (defined by KDIGO criteria) who had hemodynamic monitoring within 12 hours of AKI I. We recorded FB, urinary output (UO), fluid administered and hemodynamic parameters including mean arterial pressure (MAP) on the day of AKI I and in the following 72 hours. Logistic regression was employed to determine independent predictors of outcome.

## Results

A total of 210 patients (median age 70 years; 138 male) had hemodynamic monitoring within 12 hours of AKI I. In total, 41.5% progressed to AKI III and 43.3% died. Patients with fluid overload after the diagnosis of AKI I (FB >1 l/day; *n *= 85) had a higher rate of progression to AKI III (63.5 vs. 23.3%, respectively; *P <*0.001) and inhospital mortality (43.5 vs. 24.8%, respectively; *P *= 0.004) compared with patients with FB <1 l/day. There was no difference in the other parameters (demographics, comorbidities, severity scores, hemodynamic parameters on day of AKI I, vasopressor use), with the exception of MAP on the day of AKI I (71 vs. 74 mmHg, respectively; *P *= 0.01). In multivariate analysis after adjustment for demographics, severity scores, comorbidities and hemodynamic parameters on the day of AKI I, a higher FB was associated with an increased risk of progression to AKI III and death (odds ratio (OR) per each 1 l/day increase 2.8; *P *< 0.001) and death (OR 1.6; *P *= 0.001). Similarly, a higher amount of fluid administered was associated with an increased risk of AKI progression (OR per each 1 l/day increase 1.8; *P *= 0.011), even with adjustment for UO. There were no significant interactions between the risk of progression/death and other hemodynamic parameters. With the exception of MAP, improvements in other hemodynamic parameters in the days after AKI I diagnosis did not have a significant impact on outcome.

## Conclusion

In critically ill patients with early AKI, a positive fluid balance, induced by excessive fluid administration, is associated with an increased risk of AKI progression and death.

